# A Multitask Deep Learning Model for Predicting Myocardial Infarction Complications

**DOI:** 10.3390/bioengineering12050520

**Published:** 2025-05-14

**Authors:** Fazliddin Makhmudov, Normakhmad Ravshanov, Dilshot Akhmedov, Oleg Pekos, Dilmurod Turimov, Young-Im Cho

**Affiliations:** 1 Department of Computer Engineering, Gachon University, Sujeong-gu, Seongnam-si 13120, Republic of Korea; fazliddin12@gachon.ac.kr (F.M.);; 2 Digital Technologies and Artificial Intelligence Development Research Institute, 17A, Buz-2, Tashkent 100125, Uzbekistan

**Keywords:** cardiac rehabilitation, multitask neuron network, loss function, class imbalance, clinical factors

## Abstract

Myocardial infarction is one of the most severe forms of ischemic heart disease, associated with high mortality and disability worldwide. The accurate and reliable prediction of adverse cardiovascular events is critical for developing effective treatment strategies and improving outcomes in cardiac rehabilitation. Traditional prognostic models, such as the GRACE and TIMI scores, often lack the flexibility to incorporate a wide range of contemporary clinical predictors. Therefore, machine learning methods, particularly deep neural networks, have recently emerged as promising alternatives capable of enhancing predictive accuracy and enabling more personalized care. This study presents a multitask deep learning model designed to simultaneously address two related tasks: multidimensional binary classification of myocardial infarction complications and multiclass classification of mortality causes. The model was trained on a dataset of 1700 patients, encompassing 111 clinical and demographic features. Experimental results demonstrate high predictive accuracy and the model’s capacity to capture complex interactions among risk factors, suggesting its potential as a valuable tool for clinical decision support in cardiology. Comparative analysis confirms that the proposed multitask approach performs comparably to, or better than, conventional machine learning models. Future research will focus on refining the model and validating its generalizability in real-world clinical environments.

## 1. Introduction

Myocardial infarction (MI) is one of the most severe forms of ischemic heart disease, characterized by high patient mortality and a significant incidence of disability [1,2]. Although recent advances in diagnostics and treatment have reduced the overall fatality rate, predicting complications and mortality in individuals who have experienced MI remains a critical task in clinical practice. To address this need, the medical community—particularly cardiologists—develops and routinely uses specialized calculators or risk scales such as GRACE, ACTION, TIMI, HEART, CRUSADE, and EuroSCORE. These tools estimate the likelihood of various complications, adverse cardiac events, and patient mortality.

Researchers typically design cardiac scales by analyzing extensive clinical data. Key risk factors are identified through statistical methods, and weights are assigned to reflect their influence on the probability of specific outcomes. Traditional scales are often praised for their simplicity, interpretability, and wide acceptance in the medical community. An example is the GRACE scale, which is frequently used to assess in-hospital mortality risk in patients with acute coronary syndrome [3,4,5]. Despite these benefits, linear or simple logistic models underpinning such scales can limit flexibility and accuracy. Each scale captures a fixed set of attributes and may fail to adapt to new data.

Growing interest in machine learning (ML), especially deep neural networks, has emerged in part because these techniques can capture interactions among clinical features. ML refers to a broad class of data-driven algorithms, such as decision trees, support vector machines, and ensemble methods, which typically rely on manually selected features. In contrast, deep learning (DL) is a subfield of ML that uses multilayer neural networks capable of automatically learning complex hierarchical patterns from raw input data. This enables DL models to capture complex, non-linear interactions among clinical factors more effectively, making them particularly suitable for high-dimensional and heterogeneous medical datasets.

Modern DL approaches already play an active role in clinical research [6,7] and cardiology practice [8,9,10,11,12], and the number of related scientific publications continues to increase. One notable study [13] introduced the CSDNN model to predict mortality in patients with MI and hypertension [14]. This deep neural network addressed data imbalance in situations where mortality cases were comparatively rare. It showed improved predictive accuracy using data from the South Korean MI registry, particularly in identifying high-risk patients by shifting the decision threshold. Also, the same CSDNN network was able to predict various complications from MI, such as cardiogenic shock, pulmonary edema, and congestive heart failure, which allowed for quicker medical responses. Scientists from Taiwan [15] introduced a new deep learning model called ECG-MACE that can predict major heart problems within a year using regular ECGs, based on data from the Chang Gung Memorial Hospital.

Another investigation [16] compared an FT-Transformer architecture with a multilayer perceptron using the same South Korean MI registry. The FT-Transformer achieved higher sensitivity in mortality prediction. This study also emphasized interpretability through the SHAP method, increasing trust in neural networks for clinical applications. By modeling different scenarios based on medical records, physicians could better understand critical survival factors such as age and medication use [17].

Several publications focus on multitasking approaches that simultaneously predict complications and causes of death, thereby improving overall prognostic accuracy. An example is the use of neural networks to identify potential complications in patients after MI [18]. Other researchers [19] suggested a deep learning algorithm that can predict cardiac arrest in patients who have had a myocardial infarction while they are in the hospital, showing it is very accurate for early detection. Another study [20,21,22] used deep learning to predict long-term death rates in patients with suspected coronary artery disease, revealing that ML methods can outperform traditional statistical models.

A team [23] further developed a predictive system for myocardial infarction risk using a large clinical dataset. Their result indicated that advanced models, including boosting classifiers and convolutional neural networks (CNNs), surpassed classical decision trees (DT) and random forests (RF) by achieving high accuracy in identifying patients at risk of heart attack across different scenarios and metrics. Another group [24] aimed to develop and test a clinical risk model for in-hospital mortality in acute myocardial infarction (AMI), using the Registry-GWTG database (243,440 patients, 655 hospitals). With an in-hospital mortality rate of 4.6%, predictors such as age, heart rate, systolic blood pressure, cardiogenic shock, and troponin were especially important. Mortality varied significantly, from 0.4% in the lowest-risk group to 49.5% in the highest. Based on these experiments, the authors concluded that their model offers a valid tool for risk adjustment and stratification in AMI care. In [25], scientists suggest using a deep learning (DL) method to effectively detect myocardial infarction (MI) in two situations: one with balanced data and one without. We used an MI dataset containing 1700 medical records of MI patients.

South Korean researchers then introduced a new DAMI mortality risk stratification model [26,27] using deep learning. The DAMI model predicted both in-hospital and 12-month mortality in MI patients more accurately than existing risk assessments and other ML methods, assisting in refining rehabilitation strategies and guiding treatment decisions. Its training data included 22,875 MI patients from the KorMI registry, incorporating a broad array of demographic and laboratory variables. Validation results showed its effectiveness compared to GRACE, ACTION, and TIMI scales.

These scientific publications, along with many other studies, highlight the promise of neural networks in cardiology. Highly accurate and interpretable models for MI-related predictions enable the incorporation of multiple clinical factors, improving risk stratification and leading to more informed clinical decisions and a higher quality of care [28,29,30,31,32,33]. Nonetheless, most neural networks developed to date focus on solving individual classification or regression problems. Neural networks are typically designed with a single output layer, as they are intended to address a single specific task. In classification problems, the output layer maps the input to one of the predefined classes, whereas in regression tasks, it generates a continuous numerical prediction.

Multitask learning (MTL) architectures require more elaborate design choices, including carefully balanced loss functions and access to large, well-structured datasets. In medical applications—where class imbalance and data sparsity are common—implementing and validating MTL models is particularly challenging. As a result, the majority of published studies rely on single-task neural networks.

In more complex scenarios involving multiple outcomes or the need for insight into disease progression, researchers sometimes apply cascades or ensembles of separate neural networks. While this approach can be effective, decomposing the original problem into independent subtasks may lead to redundancy and loss of shared information. This is because breaking the original problem into separate sub-tasks usually means creating and managing several different models, which makes the process more complicated and requires more resources [15]. Moreover, this strategy can lead to redundant feature processing and prevents the models from capturing potentially important shared patterns or dependencies across tasks. Multitask learning mitigates these issues by enabling joint modeling of related outcomes within a unified architecture. Multitask deep learning offers a compelling alternative, allowing multiple related tasks to be solved jointly, potentially leveraging shared dependencies within the data. Despite its advantages, this approach remains relatively underexplored in the current literature.

Given these considerations, the present study aims to develop a multitask deep learning model designed to handle two closely related tasks: multidimensional binary classification of MI complications and multiclass classification of MI mortality causes.

We organize the rest of the paper as follows: Section 2 describes the dataset, model architecture, and methods used for training and evaluation. Section 3 presents the experimental results and model performance metrics. Section 4 discusses the implications of the findings, including comparisons with existing approaches and clinical relevance. Finally, Section 5 summarizes the conclusions and outlines direction for future research.

## 2. Materials and Methods

### 2.1. Data Description

The database of MI patients used in this study was compiled at the Krasnoyarsk Interdistrict Clinical Hospital No. 20, named after I.S. Berzon (Russia), between 1992 and 1995 [34]. Its creators originally designed it to evaluate various recognition and prediction systems.

This dataset contains comprehensive medical information on 1700 MI patients, including clinical data at admission, demographic characteristics, medical histories, laboratory and instrumental diagnostic results, therapeutic details, and more. In total, there are 111 independent variables (predictors) plus two sets of target variables. The first set covers potential MI complications—such as atrial fibrillation, supraventricular tachycardia, pulmonary edema, myocardial rupture, and other issues—which amount to 11 binary target variables. The second set looks at the reasons for deadly outcomes, like cardiogenic shock, pulmonary edema, myocardial rupture, asystole, and others, combined into one category.

Of the 1700 patients, 232 died in the intensive care unit due to various complications. Like many real-world datasets, this one can contain inconsistencies and exhibits significant class imbalance, necessitating preprocessing before building predictive models. According to the dataset’s creators [35], all 111 predictors (columns 2–112) are generally applicable for prediction. Moreover, four different time points can be used for making predictions, based on the available information:

- At hospital admission (all columns except 93–95, 100–105);
- At 24 h after admission (all columns except 94, 95, 101, 102, 104, 105);
- At 48 h after admission (all columns except 95, 102, 105);
- At 72 h after admission (all columns 2–112).

For this study, all available predictors were incorporated to train the neural network for both multivariate binary classification of complications and multiclass classification of mortality causes.

### 2.2. Multitask Neural Network Model Architecture

**Figure 1:**
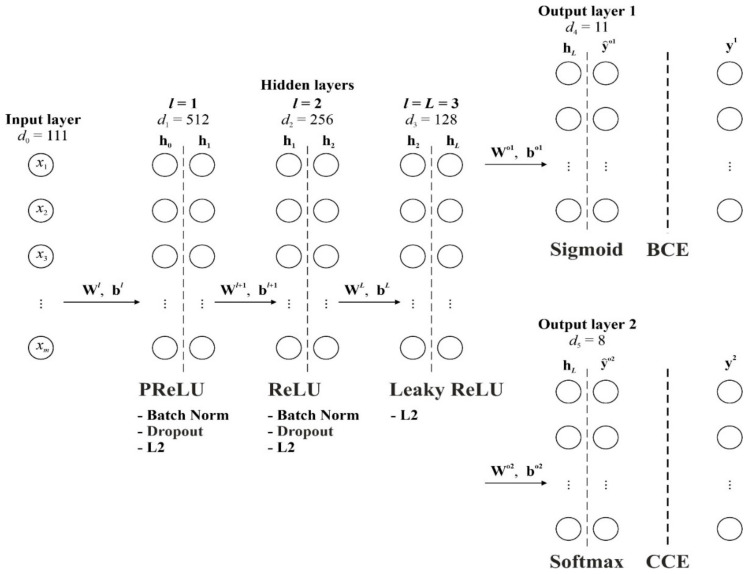
Architecture of the multitask neural network.

Let us describe the model architecture (Figure 1).

Let $X\in {\mathbb{R}}^{n\times m}$ be the feature matrix, where n is the number of patients, m is the number of features. Each patient is represented by a vector of features $x_{i}\in {\mathbb{R}}^{m}$, i=1,2,…,n.

The neural network consists of L hidden layers with the parameters $\left\{W^{l},b^{l}\right\},\;l=1,2,\ldots ,L.$ The number of hidden layers and the dimensionality of each layer $d_{l}$, are hyperparameters of the model, i.e., can be selected during the tuning process.

In our case, key hyperparameters and training settings of the multitask neural network are presented in Table 1.

The proposed multitask neural network was implemented using Python (version 3.12) and TensorFlow/Keras frameworks.

### 2.3. Forward Pass and Activation Functions

For each hidden layer l, an output value is computed:

(1) $$h^{l}=g^{l}\left(W^{l}h^{l-1}+b^{l}\right),$$

where $W^{l}\in {\mathbb{R}}^{d_{l}\times d_{l-1}}$ is the weight matrix of layer l; $b^{l}\in {\mathbb{R}}^{d_{l}}$ is the bias vector of layer l; $g^{l}$ is the activation function for layer l.

The first input $h^{0}=x_{i}$ is initialized with random values, according to the He initialization method

(2) $$W_{ij}^{l}∼N\left(0,\frac{2}{d_{l-1}}\right),$$

and the bias vector is initialized with zeros $b_{j}^{l}=0$.

First, the affine transformation

(3) $$z^{l}=W^{l}h^{l-1}+b^{l}$$

is applied, and then the batch normalization for each j-th neuron as well:

(4) $$\mu_{j}^{l}=\frac{1}{B}\sum_{i=1}^{B}z_{ij}^{l},\;{\left(\sigma_{j}^{l}\right)}^{2}=\frac{1}{B}\sum_{i=1}^{B}{\left(z_{ij}^{l}-\mu_{j}^{l}\right)}^{2},$$

In our case, the batch size is B=64.

Next, the activations are normalized

(5) $${\hat{z}}_{ij}^{l}=\frac{z_{ij}^{l}-\mu_{j}^{l}}{\sqrt{{\left(\sigma_{j}^{l}\right)}^{2}+\varepsilon }},$$

where ε is a small number to ensure stability, usually $\varepsilon =10^{-5}$.

Then, the scaling and shifting parameters are applied:

(6) $${\tilde{z}}_{ij}^{l}=\gamma_{j}^{l}{\hat{z}}_{ij}^{l}+\beta_{j}^{l},$$

which are initialized as $\gamma_{j}^{l}=1$ and $\beta_{j}^{l}=0$.

The choice of activation function depends on the specific layer and is part of the model tuning. It is known that when using only conventional activation functions like ReLU, the problem of “vanishing gradient” may arise during the training of multilayer networks, where error derivatives with respect to weights become very small as they propagate backwards through the layers. This leads to weights in the initial layers of the network being updated extremely slowly or not changing at all, which slows down or even stops the training of the entire network.

To improve learning and avoid the above problem, but without excessively increasing the computational cost, different activation functions are used here.

Parametric ReLU or PreLU for the first hidden layer (l=1)

(7) $$h_{ij}^{1}=\left\{\begin{array}{ll} {\tilde{z}}_{ij}^{1}, & {\tilde{z}}_{ij}^{1}\geq 0;\\ \alpha_{j}^{1}{\tilde{z}}_{ij}^{1}, & {\tilde{z}}_{ij}^{1}<0, \end{array}\right.$$

where $\alpha_{j}^{1}$ is trainable parameter for each neuron in the layer, initialized by small positive value, for example $\alpha_{j}^{1}=0.25$.

The ReLU for the second layer (l=2)

(8) $$h_{ij}^{2}=\left\{\begin{array}{ll} {\tilde{z}}_{ij}^{2}, & {\tilde{z}}_{ij}^{2}>0;\\ 0, & {\tilde{z}}_{ij}^{2}\leq 0. \end{array}\right.$$

And the Leaky ReLU for the third layer (l=3),

(9) $$h_{ij}^{3}=\left\{\begin{array}{ll} {\tilde{z}}_{ij}^{3}, & {\tilde{z}}_{ij}^{3}\geq 0;\\ \alpha {\tilde{z}}_{ij}^{3}, & {\tilde{z}}_{ij}^{3}<0, \end{array}\right.$$

where α≪1 is an immutable parameter with a specified value, e.g., α=0.01.

In order to prevent overfitting, the dropout method is applied to the first two hidden layers l=1 and l=2 with a neuron dropout probability $p_{0}^{l}$. For each of these two layers, a binary masking vector $r^{l}\in {\mathbb{R}}^{d_{l}}$ is created, where each element $r_{j}^{l}$ is an independent random variable for each neuron, taking values as follows:

(10) $$r_{j}^{l}=\left\{\begin{array}{ll} 1, & \mathrm{with}\;\mathrm{probability}\;p_{1}^{l};\\ 0, & \mathrm{with}\;\mathrm{probability}\;p_{0}^{l}, \end{array}\right.$$

where the probabilities $p_{1}^{l}=0.6$ and $p_{0}^{l}=0.4$ were selected experimentally.

Then, the obtained mask is applied to the activations

(11) $$h_{ij}^{l}=\frac{r_{j}^{l}}{p_{1}^{l}}h_{ij}^{l}.$$

Here, division by $p_{1}^{l}$ is necessary to preserve the mathematical expectation of activations.

According to the problem statement, the proposed model contains two output layers. The first output layer is for multivariate binary classification of k=11 MI complications. Here, the sigmoid activation function was used:

(12) $${\hat{y}}_{ij}^{o1}=\sigma \left(\sum_{s=1}^{d_{L}}W_{js}^{o1}h_{is}^{L}+b_{j}^{o1}\right),j=1,2,\ldots ,k.$$

The second output layer for the multiclass classification of C=8 causes of lethal outcomes. At this layer, the softmax activation is used:

(13) $${\hat{y}}_{ij}^{o2}=\frac{\exp\left(\sum_{s=1}^{d_{L}}W_{js}^{o2}h_{is}^{L}+b_{j}^{o2}\right)}{\sum_{c=1}^{C}\exp\left(\sum_{s=1}^{d_{L}}W_{cs}^{o2}h_{is}^{L}+b_{c}^{o2}\right)},j=1,2,\ldots ,C.$$

### 2.4. Backpropagation, Class Imbalance and Loss Functions

Before describing backpropagation step, it is important to highlight a key point. Class imbalance is often present in medical data. The dataset of patients with MI which was used for training is no exception. To eliminate the class imbalance, we perform oversampling of minority classes up to 50% of the number in the majority class. Also, in order to avoid overfitting, the L2-regularization is applied here along with the dropout to ensure that the model adequately handle new data.

The loss function of the first classifier is evaluated using the binary cross-entropy

(14) $$L_{1}=-\frac{1}{n\times k}\sum_{i=1}^{n}\sum_{j=1}^{k}\left[y_{ij}^{o1}\ln\left({\hat{y}}_{ij}^{o1}\right)+\left(1-y_{ij}^{o1}\right)\ln\left(1-{\hat{y}}_{ij}^{o1}\right)\right],$$

where $y_{ij}^{o1}\in \left\{0,1\right\}$ is the true value of the presence or absence of j-th complication for the i-th patient; ${\hat{y}}_{ij}^{o1}$ is the predicted probability of j-th complication for the i-th patient.

The categorical cross-entropy is used as the loss function for the second classifier, which predicts the causes of fatal outcomes:

(15) $$L_{2}=-\frac{1}{n}\sum_{i=1}^{n}\sum_{j=1}^{C}y_{ij}^{o2}\ln\left({\hat{y}}_{ij}^{o2}\right),$$

where $y_{ij}^{o2}\in \left\{0,1\right\}$ is the true class label, $y_{ij}^{o2}=1$ if the i-th patient belongs to j-th class, otherwise $y_{ij}^{o2}=0$; ${\hat{y}}_{ij}^{o2}$ is the predicted probability of that i-th patient belongs to j-th class.

The overall loss function is the sum these two loss functions, taking into account the regularization:

(16) $$L=\alpha L_{1}+\beta L_{2}+R,$$

where α=3.0 и β=1.0 are the hyperparameters determining the contribution of each task, whose values were selected experimentally; R is the regularization term

(17) $$R=\frac{\lambda }{2}\left(\sum_{l=1}^{L}\|W^{l}{\|}_{F}^{2}+\|W^{o1}{\|}_{F}^{2}+\|W^{o2}{\|}_{F}^{2}\right),$$

where λ=0.001 is the regularization factor; ${\left\|\cdot \right\|}_{F}$ is the Frobenius norm.

The gradients of the loss function L over all model parameters are calculated during backpropagation.

For MI complications, first the output error is calculated

(18) $$\delta_{ij}^{o1}={\hat{y}}_{ij}^{o1}-y_{ij}^{o1}.$$

Then, the gradients with respect to the parameters are calculated

(19) $$\frac{\partial L}{\partial W_{js}^{o1}}=\frac{\alpha }{B}\sum_{i=1}^{B}\delta_{ij}^{o1}h_{is}^{L}+\lambda W_{js}^{o1};\frac{\partial L}{\partial b_{j}^{o1}}=\frac{\alpha }{B}\sum_{i=1}^{B}\delta_{ij}^{o1}.$$

Similar calculations are performed for mortality

(20) $$\delta_{ij}^{o2}={\hat{y}}_{ij}^{o2}-y_{ij}^{o2};\;\frac{\partial L}{\partial W_{js}^{o2}}=\frac{\beta }{B}\sum_{i=1}^{B}\delta_{ij}^{o2}h_{is}^{L}+\lambda W_{js}^{o2};\frac{\partial L}{\partial b_{j}^{o2}}=\frac{\beta }{B}\sum_{i=1}^{B}\delta_{ij}^{o2}.$$

Next, the gradients of the hidden layers are computed. For the hidden layers l=L,L−1,…,1 starting from L=3, the total error is calculated as:

(21) $$\delta_{ij}^{L}=\left(\sum_{s=1}^{k}\delta_{is}^{o1}W_{sj}^{o1}+\sum_{c=1}^{C}\delta_{ic}^{o2}W_{cj}^{o2}\right)g^{\prime }\left({\tilde{z}}_{ij}^{L}\right),$$

where s is the summation index over the complication classes from 1 to k; c is the summation index over the mortality classes from 1 to C; $g^{\prime }\left({\tilde{z}}_{ij}^{L}\right)$ is the derivative of Leaky ReLU for the third hidden layer

(22) $$g^{\prime }\left({\tilde{z}}_{ij}^{L}\right)=\left\{\begin{array}{ll} 1, & \mathrm{if}\;{\tilde{z}}_{ij}^{L}\geq 0;\\ \alpha , & \mathrm{if}\;{\tilde{z}}_{ij}^{L}<0. \end{array}\right.$$

Next, the gradients with respect to the parameters are calculated

(23) $$\frac{\partial L}{\partial W_{js}^{L}}=\sum_{i=1}^{B}\delta_{ij}^{L}h_{is}^{L-1}+\lambda W_{js}^{L};\frac{\partial L}{\partial b_{j}^{L}}=\sum_{i=1}^{B}\delta_{ij}^{L}.$$

The process is repeated for the second l=2 and first l=1 hidden layers, taking into account the neurons dropout $\delta_{ij}^{l}=\delta_{ij}^{l}\left(r_{j}^{l}/p_{1}^{l}\right)$ and batch normalization, where gradients have the following form:

(24) $$\frac{\partial L}{\partial \gamma_{j}^{l}}=\sum_{i=1}^{B}\delta_{ij}^{l}{\tilde{z}}_{ij}^{l};\frac{\partial L}{\partial \beta_{j}^{l}}=\sum_{i=1}^{B}\delta_{ij}^{l}.$$

It should be noted that the complete formulation of backpropagation through Batch Normalization is not given here, as it is quite complex and involves multiple steps. It was omitted because the implementation utilized built-in algorithms from Keras and TensorFlow libraries.

Also, the parameters of the PReLU are taken into account:

(25) $$\frac{\partial L}{\partial \alpha_{j}^{l}}=\sum_{i=1}^{B}\delta_{ij}^{l}{\hat{z}}_{ij}^{l}\cdot I\left({\hat{z}}_{ij}^{l}<0\right),$$

where I is the indicator function.

### 2.5. Parameter Updates with Adam Optimizer

For adaptive parameter updating, Adam optimizer [36] is used. According to the algorithm, the moment update expressions are

(26) $$m_{\theta }=\beta_{1}m_{\theta }+(1-\beta_{1})\frac{\partial L}{\partial \theta };\;v_{\theta }=\beta_{2}v_{\theta }+(1-\beta_{2}){\left(\frac{\partial L}{\partial \theta }\right)}^{2},$$

where θ is any model parameter (weights, biases, normalization parameters, activation function parameters, etc.); $\beta_{1}$ and $\beta_{2}$ are the exponential decay coefficients of the moments with predefined values $\beta_{1}=0.9$ и $\beta_{2}=0.999$. The initialization of moments $m_{0}=0$ and $v_{0}=0$.

Bias correction is performed as follows:

(27) $${\hat{m}}_{\theta }=\frac{m_{\theta }}{1-\beta_{1}^{t}};\;{\hat{v}}_{\theta }=\frac{v_{\theta }}{1-\beta_{2}^{t}},$$

and parameter updating as:

(28) $$\theta_{t+1}=\theta_{t}-\eta \frac{{\hat{m}}_{\theta }}{\sqrt{{\hat{v}}_{\theta }}+\epsilon },$$

where t is the current iteration number; η=0.0005 is the learning rate; ϵ is a small number to prevent division by zero, usually set to $1\times 10^{-8}$.

The stopping criteria for the iterative process are the conditions for reaching the maximum number of epochs; the absence of improvements on the validation set over a specified number of epochs (early stopping); and achieving the required value of the loss function or quality metric.

## 3. Results

*Model Performance Evaluation*

**Figure 2:**
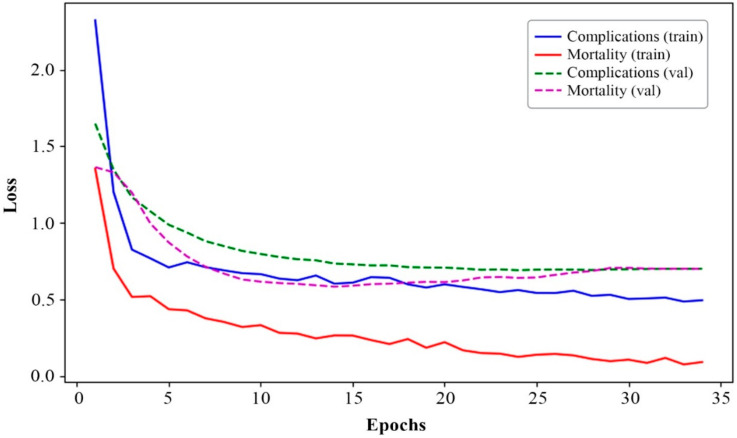
Changes in loss for complication and mortality classes over epochs.

**Figure 3:**
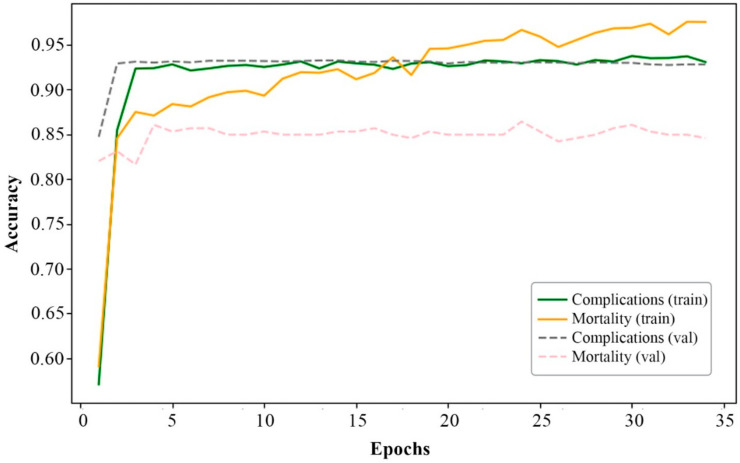
Changes in model accuracy for complication and mortality classes over epochs.

**Figure 4:**
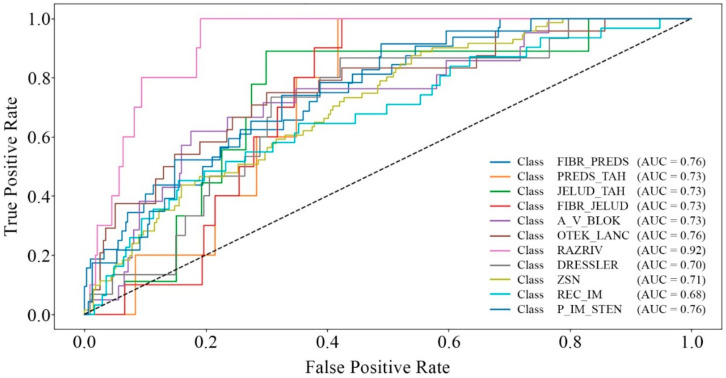
ROC curves for complication classes.

**Figure 5:**
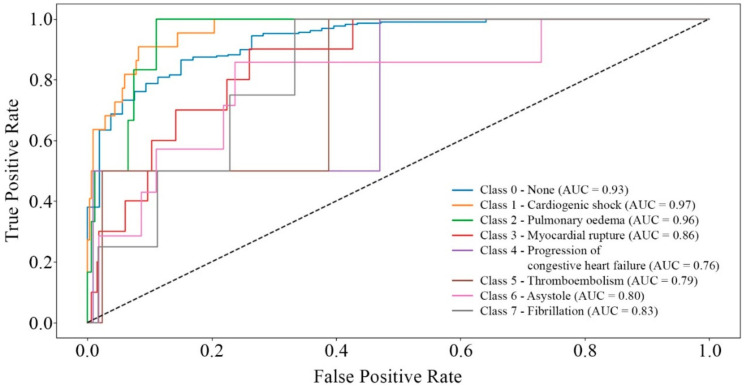
ROC curves for mortality classes.

Figure 2, Figure 3, Figure 4 and Figure 5 present the results of the model performance evaluation. The provided metric graphs help us see how well the model does in identifying MI complications and the different causes of mortality.

The curves in Figure 2 illustrate the changes in loss for complication and mortality classes over epochs for the training (train) and validation (val) datasets. As observed, there is a sharp decrease in loss during the first few epochs, followed by stabilization at a low level. This indicates that the model learns efficiently in the early stages and gradually approaches convergence by epoch 25.

Additionally, the loss curve for mortality causes decreases more rapidly and stabilizes at a lower level compared to complications, suggesting better convergence of the model for mortality prediction.

Figure 3 illustrates the changes in accuracy over epochs for complication and mortality classes, similar to Figure 2, for both the training (train) and validation (val) datasets. As seen in Figure 3, accuracy increases rapidly during the initial epochs and then remains consistently high, reaching approximately 0.9. This trend indicates effective model learning and its ability to maintain high accuracy throughout training.

However, as observed in Figure 2 and Figure 3, as well as in Table 1, which presents the metric values, the curves plateau around epoch 34. At this point, validation performance starts to deteriorate significantly, indicating the onset of overfitting.

Overall, the results in Figure 2 and Figure 3, as well as Table 2, indicate successful model training with satisfactory convergence for both prediction tasks and most classes—77% for complications and 86% for mortality.

Figure 4 shows that the area under the curve (AUC) values vary across different classes, ranging from 0.68 to 0.92. This indicates that the model performs well or satisfactorily in distinguishing most complication classes. However, its accuracy is noticeably lower for certain cases, such as “REC_IM” (myocardial infarction recurrence) with an AUC of 0.68.

The ROC curves presented in Figure 5 illustrate the AUC values for different mortality classes. Unlike Figure 4, these values are significantly higher, reaching a maximum of 0.97, which indicates better model performance in predicting mortality compared to complications. However, variations in accuracy between different mortality causes are still observed, similar to the case of MI complications.

Based on the analysis of the results (Figure 2, Figure 3, Figure 4 and Figure 5), it is evident that adding more training data and implementing additional class-balancing techniques could further improve model performance. Additionally, fine-tuning hyperparameters such as learning rate and regularization settings could enhance prediction quality.

## 4. Discussion

### 4.1. Comparison with Other ML Models

Since this dataset has been used in multiple studies by researchers from various countries, it is possible to compare the quality of the proposed model with machine learning algorithms developed in those studies.

For example, in study [37], the authors focused on predicting the risk of MI complications within the first few hours after patient hospitalization. Their interest stemmed from the fact that the initial hours of hospitalization are typically the most critical for patient survival. Notably, the authors of [29] did not utilize deep learning models but instead applied commonly used machine learning algorithms.

Table 3 presents the accuracy results of classifiers tested on this dataset. It is important to emphasize that the authors addressed only a single task—predicting complications.

The same study also provides separate results evaluating the performance of the XGBoost classifier combined with various synthetic data generation methods aimed at addressing class imbalance in the dataset. This includes a method developed by the authors themselves (Table 4).

Similar studies have been conducted by other researchers. For example, in study [38], a method for predicting patient mortality in MI cases was proposed based on the same dataset [35]. The authors used the Cox regression model to estimate mortality risk and identify key factors influencing patient survival. Reddy L. and Thangam S. [39] implemented several machine learning algorithms with different sampling methods to predict a specific complication—MI recurrence. Their findings indicated that the SVM classifier demonstrated the highest accuracy. A team of Indian researchers led by Joshi A. applied the mutual information technique to select the most informative features, identifying 20 key predictors [40]. Using the SVM classifier, they achieved an F1 score of 90.29%, focusing specifically on predicting adverse MI outcomes.

Table 5 presents the performance evaluation results of machine learning models developed by these authors, tested on the Krasnoyarsk Interdistrict Clinical Hospital dataset [35].

As seen in Table 2, the performance of the developed multitask neural network model is only slightly lower than that of commonly used machine learning algorithms. It is important to note that in the study by Newaz A. and other authors [37], the classifiers were applied to solve only a single prediction task.

The higher accuracy of the XGBoost algorithm (Table 3) was achieved primarily through aggressive data resampling techniques to mitigate class imbalance in complications. However, such approaches can reduce the model’s true predictive capability by artificially balancing the dataset rather than improving its generalization ability.

In our case, because of pronounced class imbalance (for certain complications and mortality causes), we used oversampling of minority classes up to 50% of the majority class size. Additionally, we experimented with SMOTE, ADASYN, and other sampling strategies, but the simpler oversampling approach performed well in combination with the multitask scheme. Weighted loss functions were also tested, but oversampling yielded satisfactory improvements in practice.

Based on a comparative analysis of the performance of complication and mortality prediction models (Table 4) developed by various authors, the proposed multitask neural network model demonstrates comparable effectiveness. In certain cases (Figure 2, Figure 3, Figure 4 and Figure 5), it even surpasses traditional methods in accuracy. Additionally, unlike other models, it can solve two tasks simultaneously without excessive data resampling, preserving the dataset’s natural distribution.

### 4.2. Clinical Interpretation and Feature Importance

Permutation importance analysis was conducted to quantify the impact of each clinical predictor on model performance. Shuffling key features such as age, systolic blood pressure, and left ventricular ejection fraction resulted in a 7–10% decrease in overall model accuracy, highlighting their substantial importance for prediction outcomes. Additional influential variables included blood glucose level and heart rate, each associated with a 3–5% reduction in accuracy when permuted. These findings are consistent with previous studies [28,29,37], where age and cardiac function indices were identified as major risk factors for adverse MI outcomes and mortality.

Moreover, our multitask model identified a broader set of clinically relevant variables compared to single-task models reported in earlier works. For example, Newaz et al. [37] and Kim et al. [16] also emphasized the predictive value of age and blood pressure but did not analyze the simultaneous effect of these features on both complications and mortality. By jointly modeling both tasks, our approach enables more comprehensive risk stratification and highlights the shared and unique contributions of clinical predictors across outcomes.

From a clinical standpoint, it is also important to investigate whether certain complications share correlated predictors. For instance, in some patients, a risk factor for pulmonary edema may also be relevant for cardiogenic shock. A multitask model can leverage such shared information, potentially explaining why it outperforms single-task classifiers that handle each outcome independently.

Although permutation importance provides useful insights, further analysis using more advanced interpretability methods, such as SHAP, may help clarify complex interactions among predictors. This is planned for future work.

## 5. Conclusions

This study yields the following key conclusions.

From a theoretical perspective, the contribution of this research lies in the development and implementation of a multitask neural network architecture tailored for the simultaneous prediction of multiple clinical outcomes, as well as the integration of permutation-based feature importance analysis to enhance model interpretability. The approach leverages a combination of advanced activation functions (PReLU, ReLU, LeakyReLU) to optimize learning and incorporates oversampling strategies to address class imbalance, ensuring robust model performance. Our analysis demonstrates that the proposed model achieves performance comparable to, and in some cases surpasses, traditional machine learning methods commonly used for similar tasks. Notably, the model effectively handles class imbalance—a common challenge in medical datasets collected in real-world clinical practice.

From a practical perspective, the model provides a robust and versatile tool for individualized risk stratification in MI patients, supporting more informed clinical decision-making. The model is already integrated into a telemedicine system for remote monitoring and management of cardiac rehabilitation https://recardio.uz (accessed on 17 February 2025). It serves as a supplementary tool for predicting adverse cardiovascular events and assessing patient severity, thereby assisting clinicians in selecting individualized rehabilitation programs, including recommendations for physical activity and pharmacological management for MI patients. Its ability to process heterogeneous clinical and demographic data, as well as to simultaneously predict multiple outcomes, highlights its potential for integration into real-world decision support systems in cardiology.

Overall, the results of this study confirm the promising potential of deep learning models in cardiology, particularly for predicting complication risks and mortality in MI patients, thereby supporting more accurate and personalized clinical decision-making.

Future work will focus on employing deeper interpretability methods, such as SHAP or LIME, and on evaluating the model’s generalizability and stability across different populations [41,42]. The promising results indicate that multitask neural networks can serve as an effective alternative to single-task or multi-model approaches for predicting MI complications and mortality.

## Figures and Tables

**Table 1:** Key hyperparameters and training settings of the multitask neural network.

Parameter	Value/Description
Input features	$d_{0}=m$ = 111 clinical and demographic variables
Hidden layers	3
Layer 1	$d_{1}=512$, PReLU, L2 regularization (λ=0.001), BatchNormalization, Dropout (rate = 0.4)
Layer 2	$d_{2}=256$, ReLU, L2 regularization (λ=0.001), BatchNormalization, Dropout (rate = 0.4)
Layer 3	$d_{3}=128$, LeakyReLU (α=0.01), L2 regularization (λ=0.001)
Output layers	2
Complications	$d_{4}=11$, binary classification, Sigmoid activation
Mortality causes	$d_{5}=8$, multiclass classification, Softmax activation
Optimizer	Adam (learning rate = 0.0005)
Batch size	64
Epochs	E = 50 (with early stopping, patience = 10)
Loss function (complications)	Binary cross-entropy
Loss function (mortality)	Sparse categorical cross-entropy
Loss weights	Complications: 3.0; Mortality: 1.0
Class imbalance handling	Random oversampling of minority classes up to 50% of the majority class (for complications)
Feature scaling	StandardScaler (zero mean, unit variance)
Validation strategy	Train/test split: 80/20; 20% of training set used for validation during training
Regularization	L2 regularization (λ=0.001); Dropout (rate = 0.4 on first two hidden layers)
Callbacks	EarlyStopping (patience = 10, restore best weights), ReduceLROnPlateau (factor = 0.1, patience = 5)
Evaluation metrics	Binary accuracy, AUC (for complications); Categorical accuracy (for mortality causes)

**Table 2:** Metric values at epoch 34 for training and validation datasets.

Metrics		Training Set	Validation Set	Weighted and Averaged Values over Epochs
Loss	Complications	0.5080	0.7002	0.6626
	Mortality	0.0874	0.7033	0.4289
	Overall loss	1.1593	1.8873	1.7132
Accuracy	Complications	0.9347	0.9278	0.7799
	Mortality	0.9732	0.8529	0.8617
AUC	Complications	0.9087	0.7847	0.9286

**Table 3:** Accuracy of machine learning algorithms (%) for predicting MI complications at the time of hospital admission [37].

KNN	SVM	LR	NB	RF	XGBoost	AdaBoost
85.9724	86.2317	86.4902	20.75	91.1433	91.3370	90.1098

**Table 4:** Accuracy of the XGBoost classifier (%) combined with different class-balancing methods for MI complications [37].

SMOTE	ADASYN	RUS	Tomek-Link	ENN	Weighted XGBoost	This Model
91.1431	91.4009	81.9014	90.8850	90.4317	91.5311	91.9843

**Table 5:** Performance metrics of models developed by various authors [37].

Studies	Metrics		
	Accuracy (%)	Sensitivity (%)	ROC-AUC (%)
Newaz A. [37]	91.98	65.03	80.88
Farah C. [38]	87.33	25.00	61.67
Reddy L. [39]	84.87	43.98	68.04
Joshi A. [40]	86.62	15.08	57.16

## Data Availability

The original contributions presented in this study are included in the article. Further inquiries can be directed to the corresponding author.
